# The effect of climbing chalk powder on the infectivity of human coronavirus OC43

**DOI:** 10.1111/lam.13466

**Published:** 2021-03-03

**Authors:** L. Owen, K. Laird, M. Shivkumar

**Affiliations:** ^1^ Infectious Disease Research Group The Leicester School of Pharmacy De Montfort University Leicester UK

**Keywords:** calcium carbonate, climbing chalk, human coronavirus OC43, infectivity, magnesium carbonate, TCID_50_

## Abstract

There does not appear to be any studies in the published literature on the stability of SARS‐CoV‐2 in climbing chalk powder (magnesium carbonate and/or calcium carbonate), which has been hypothesized to pose a potential risk of fomite transmission of coronavirus disease 2019 (COVID‐19) within climbing gyms. The aim of this study was to determine the infectivity of a model human coronavirus HCoV‐OC43 in the presence of climbing chalk powder on a dry plastic surface. The stability of HCoV‐OC43 on a plastic surface dusted with climbing chalk powders (magnesium carbonate, calcium carbonate or a blended chalk) was determined by titration on BHK‐21 fibroblast cells. No chalk and no virus controls were included. HCoV‐OC43 was stable on the plastic surface for 48 h. The stability of HCoV‐OC43 was significantly (*P* ≤ 0·05) reduced in the presence of magnesium carbonate, calcium carbonate and the chalk blend; the infectivity was reduced by ≥2·29 log_10_ 50% tissue culture infective dose (TCID_50_) immediately upon on contact and by ≥2·46 log_10_ TCID_50_ within 1 h of contact. These findings suggest that the infectivity of coronaviruses is reduced by climbing chalk, limiting the risk of potential fomite transmission.

## Graphical abstract

**Significance and Impact of the Study:** The emergence of the coronavirus disease 2019 (COVID‐19) pandemic has resulted in the need for increased infection control measures in public settings. There does not appear to be any published research on the survival of SARS‐CoV‐2 or other coronaviruses in the presence of climbing chalk, which could pose a potential risk of fomite transmission within climbing gyms. This study demonstrates that the infectivity of a model human coronavirus HCoV‐OC43 is significantly (*P* ≤ 0·05) reduced in the presence of climbing chalk powders compared to a plastic surface, suggesting that the fomite transmission risk of climbing chalk powders is limited.

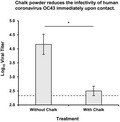

## Introduction

The emergence of severe acute respiratory syndrome coronavirus 2 (SARS‐CoV‐2) and subsequent development of the coronavirus disease 2019 (COVID‐19) pandemic has led to the widespread implementation and promotion of public health interventions, such as social distancing, closure of nonessential businesses, enhanced disinfection and handwashing to limit transmission of COVID‐19 (Hartley and Perencevich 2020; Prather *et al*. 2020). Although COVID‐19 is predominantly transmitted by respiratory droplets, it has been hypothesized that indirect transmission through contact with fomites could also occur because SARS‐CoV‐2, similarly to other coronaviruses, can remain infectious on dry surfaces for several hours (Pastorino *et al*. 2020).

It has been speculated that recreational climbing gyms could be high‐risk environments for the transmission of SARS‐CoV‐2. In particular, concerns have been raised regarding potential fomite transmission by the use of shared climbing holds which are not cleaned between users in common practice (Debenham and Reynolds 2020). SARS‐CoV‐2 has been shown to remain infectious on plastic for around three days (Van Doremalen *et al*. 2020) to seven days (Chin *et al*. 2020), indicating that SARS‐CoV‐2 could persist on plastic climbing holds.

Another speculated potential source for the transmission of COVID‐19 within climbing gyms is climbing chalk, which is applied to the hands of climbers to improve grip on holds (Bacon *et al*. 2018). Climbing chalk has been observed to deposit onto climbing holds during use (Li *et al*. 2001) and as such has been hypothesized to serve as another potential environmental reservoir of SARS‐CoV‐2. Climbing chalk powder is primarily comprised of magnesium carbonate (Li *et al*. 2001), with calcium carbonate present as an impurity or filler. There does not appear to be any studies in the published literature on the stability of SARS‐CoV‐2 or other coronaviruses in climbing chalk powder and therefore the risk of fomite transmission in this context is not well understood.

In this study, human coronavirus (HCoV‐OC43), a common causative agent of upper respiratory tract infections in humans (Liu *et al*. 2020) was used as a model for SARS‐CoV‐2 to determine stability of coronaviruses in climbing chalk powder. HCoV‐OC43 is in the same genus as SARS‐CoV‐2 (*Betacoronavirus*) and has a similar overall structure; both possess a single‐stranded positive‐sense RNA genome and are enclosed in a lipid envelope with spike proteins protruding from the surface (Chen *et al*. 2020). Previous studies suggest that the stability of SARS‐CoV‐2 in the environment is likely to be within the same order of magnitude of other coronaviruses (Aboubakr *et al*. 2020), suggesting that HCoV‐OC43 could useful as a model for SARS‐CoV‐2 in environmental stability experiments. For example, Van Doremalen *et al*. (2020) reported that the half‐lives of SARS‐CoV‐2 and SARS‐CoV were similar on inanimate objects including copper (0·8 *vs* 1·4 h), stainless steel (5·6 *vs* 4·2 h) and plastic (6·8 *vs* 7·6 h).

The aim of this study was to determine the stability of a model human coronavirus (HCoV‐OC43) in the presence of climbing chalk powder on a dry plastic surface.

## Results and discussion

HCoV‐OC43 remained relatively stable on the plastic surface without chalk, with 3·74 log_10_ 50% tissue culture infectious dose (TCID_50_) sample^−1^ infectious virus being detected on the surface after 1 h and ≤2·68 ± 0·35 after 24 h (Table 1). No significant (*P* ≤ 0·05) decrease in infectious viral titre was observed until 48 h incubation, where no infectious virus was detected (Table 1). These results are in line with previous studies conducted on the survival of SARS‐CoV‐2 on plastic surfaces. Van Doremalen *et al*. (2020) recovered 2·5 log_10_ ml^−1^ SARS‐CoV‐2 from plastic surfaces after 24 h incubation at room temperature (initial inoculum, 5 log_10_ ml^−1^), and after 48–72 h, only 0·5–1·0 log_10_ ml^−1^ was detected. No infectious virus was recovered from the no virus controls.

**Table 1:** Stability of HCoV‐OC43 on a PVC PUR surface (*n* = 3, mean ± standard error of the mean)

Time	Log_10_ TCID_50_ per sample (Log_10_ reduction from initial inoculum*)
0 min	4·16 ± 0·36 (0·63)
1 min	4·30 ± 0·03 (0·49)
5 min	4·25 ± 0·44 (0·54)
10 min	4·08 ± 0·25 (0·71)
30 min	4·25 ± 0·22 (0·54)
1 h	3·74 ± 0·35 (1·05)
6 h	3·91 ± 0·63 (0·88)
24 h	≤2·68 ± 0·35 (≥2·11)
48 h	†(≥2·46)
72 h	†(≥2·46)

* The initial inoculum was 4·79 log_10_ TCID_50_ per sample.

^†^ Below detection limit (2·33 log_10_ TCID_50_ per sample). Where one or more samples reached the detection limit of the assay (2·33 log_10_ TCID_50_ per sample) the number of infectious virus is expressed as ≤log_10_ TCID_50_.

The infectivity of HCoV‐OC43 was significantly (*P* ≤ 0·05) reduced by magnesium carbonate, calcium carbonate and the blended chalk (Table 2) compared to the no chalk control at 0 min, which was only reduced by 0·63 log_10_ TCID_50_ (Table 1). Magnesium carbonate and the chalk blend both reduced HCoV‐OC43 by ≥2·29 log_10_ TCID_50_ immediately on contact, with ≤2·50 log_10_ TCID_50_ per sample infectious virus being detected (Table 2). Calcium carbonate chalk reduced HCoV‐OC43 to a lesser extent, by 2·17 log_10_ TCID_50_.

**Table 2:** Stability of HCoV‐OC43 on a PVC PUR surface in the presence of climbing chalk powders (*n* = 3, mean ± standard error of the mean)

Time	Log_10_ TCID_50_ per sample (Log_10_ reduction from initial inoculum*)		
	Magnesium carbonate	Calcium carbonate	Blended chalk
0 min	≤2·50 ± 0·17 (≥2·29)	2·83 ± 0·09 (≥2·18)	≤2·50 ± 0·17 (≥2·29)
1 min	≤2·48 ± 0·08 (≥2·31)	≤2·46 ± 0·07 (≥2·55)	≤2·40 ± 0·07 (≥2·39)
5 min	†(≥2·46)	≤2·61 ± 0·19 (≥2·40)	†(≥2·46)
10 min	≤2·48 ± 0·15 (≥2·31)	2·78 ± 0·18 (≥2·23)	†(≥2·46)
30 min	≤2·41 ± 0·08 (≥2·38)	≤2·45 ± 0·12 (≥2·56)	≤2·40 ± 0·07 (≥2·39)
1 h	†(≥2·46)	≤2·45 ± 0·12 (≥2·56)	†(≥2·46)

* The initial inoculum was 4·79 log_10_ TCID_50_ per sample except for calcium carbonate, where the inoculum was 5·01 log_10_ TCID_50_ per sample.

^†^ Below detection limit (2·33 log_10_ TCID_50_ per sample). Where one or more samples reached the detection limit of the assay (2·33 log_10_ TCID_50_ per sample) the number of infectious virus is expressed as ≤log_10_ TCID_50_ per sample.

In this study, infectious virus was recovered from the chalk by resuspension in PBS and centrifuging to remove excess chalk. This method was employed to minimize the cytotoxicity of the chalk and allow syringe filtration of the viral supernatant to avoid bacterial contamination. In order to validate the recovery method, the distribution of viral RNA between the supernatant and the chalk pellet after 0 min contact were quantified by quantitative reverse transcription PCR (RT‐qPCR). A significantly greater (*P* ≤ 0·05) number of viral RNA was present in the magnesium carbonate supernatant compared to the pellet, where 80·3 ± 2·4% of the total RNA was recovered in the supernatant compared to the pellet. There was no significant difference (*P* > 0·05) in the recovery of HCoV‐OC43 between calcium carbonate and blended chalk supernatants and pellets, with 50·5 ± 16·4% and 39·4 ± 9·7% of the total viral RNA being recovered in the respective supernatants compared to the pellets. Although a proportion of the viral RNA was lost within the chalk pellet, removal of excess chalk could not be avoided due to cytotoxicity of the chalk against the mammalian cell lines. These results do not change the observations of an overall loss in infectivity of HCoV‐OC43 on exposure to the chalk; theoretically if virus from the pellet was also recovered, the maximum increase in infectivity in the blended chalk condition at 0 min would be to 2·9 log_10_ TCID_50_ per sample (from ≤2·5 log_10_ TCID_50_ per sample) assuming 39·4% recovery, which still results in approximately a 2 log_10_ reduction. It is important to note that detection of nucleic acids does not distinguish between infective and inactive virus and therefore it was not possible to ascertain the TCID_50_ sample^−1^ of HCoV‐OC43 within the pellet. Moreover the behaviour of the virus within the pellet would also be in line with those in the supernatant and therefore the majority are likely be noninfectious.

The number of infectious virus remained close to or below the detection limit (2·33 log_10_ TCID_50_ per sample) up to 30 min contact with magnesium carbonate and the chalk blend. No infectious virus was detected after 1 h in the presence of chalk, indicating that ≤2·33 log_10_ TCID_50_ infectious virus was present. The infectious viral titre did not change significantly (*P* > 0·05) over the course of 1 h upon contact with all three chalks. One limitation of this study is the relatively low sensitivity of the infectivity assay, where the limit of detection was 2·33 log_10_ TCID_50_ per sample. This can be attributed to cytotoxicity of the chalk supernatants upon BHK‐21 cells, thus requiring a greater level of dilution to quantify infectious virus.

The results of this study indicate that human coronavirus HCoV‐OC43 is unlikely to persist for extended periods of time within climbing chalk powders, and therefore the risk of fomite transmission is limited. There do not appear to be any studies in the published literature on the antiviral activity of climbing chalk powders. Magnesium carbonate (1 mg ml^−1^ suspension) was previously reported to inhibit the growth of *Staphylococcus epidermidis*, which was in part attributed to its alkalinity (Welch *et al*. 2016). Further research is required to determine the mechanism by which HCoV‐OC43 is inhibited by climbing chalk powders. The inactivation of HCoV‐OC43 could potentially be attributed to desiccation of the virus, due to the hygroscopic nature of magnesium carbonate and calcium carbonate, and/or the alkalinity. Desiccation has been hypothesized to lead to phase changes, peroxide formation and other reactions within the lipid envelope of coronaviruses, leading to denaturation (Casanova *et al*. 2010) and thereby preventing interaction with host cells. In accordance, previous research has indicated that HCoV‐OC43 is more stable in suspension compared to dry conditions, remaining infectious for 2 to 3 days suspended in culture media compared to less than three hours dried onto aluminium, sponge or latex gloves; a similar pattern was also observed for HCoV‐229E (Sizun *et al*. 2000). Another potential hypothesis is that binding of HCoV‐OC43 to fine chalk particles may prevent attachment to and/or entry into the cells, thus rendering the virus noninfectious.

The initial viral titre used in this study (~5 log_10_ TCID_50_ per ml) is in line with that of similar studies measuring the environmental stability of coronaviruses (Sizun *et al*. 2000; Lai *et al*. 2005; Van Doremalen *et al*. 2020). There is limited published research on the quantity of infectious virus that is shed by COVID‐19 patients and therefore a realistic infectious viral load for contaminated surfaces has not been fully ascertained. Previous work on influenza demonstrated that the infectious viral load of one cough was 5 plaque‐forming units ml^−1^ (Lindsley *et al*. 2010), suggesting that 5 log_10_ TCID_50_ per ml may be greater than that encountered under realistic conditions. However, the use of a 5 log_10_ TCID_50_ per ml inoculum allows viral titre reductions to be monitored above the detection limit of this assay, and can therefore be used to infer the viral stability in the presence of chalk under real life conditions.

Overall, the infectivity of HCoV‐OC43 is reduced by ≥2·29 log_10_ TCID_50_ immediately on contact with magnesium carbonate, calcium carbonate and a blended chalk and by ≥2·46 log_10_ TCID_50_ within 1 h contact. HCoV‐OC43 reduces naturally on the plastic surface over time, decreasing by 0·63 log_10_ TCID_50_ immediately on contact and 1·05 log_10_ TCID_50_ within one hour, which is significantly less than on contact with the chalk. This suggests that coronaviruses such as HCoV‐OC43 or SARS‐CoV‐2 are likely inactivated by climbing chalk, limiting the risk of potential fomite transmission. Further research into the stability of SARS‐CoV‐2 in climbing chalk would enhance our understanding of COVID‐19 transmission risks.

## Materials and Methods

### Micro‐organisms and cell lines

HCT‐8 epithelial cells (ECACC 90032006) were cultured at 37°C with 5% CO_2_ in RPMI‐1640 (Lonza, Basel, Switzerland) culture medium supplemented with 10% fetal bovine serum (FBS; HyClone, Logan, UT), 100 IU ml^−1^ penicillin and 100 *μ*g ml^−1^ streptomycin (Lonza, Basel, Switzerland). BHK‐21 fibroblast cells (clone 13; ECACC 85011433) were cultured in Dulbecco's Modified Eagle Medium (DMEM; Lonza, Basel, Switzerland) supplemented with 10% FBS, 100 IU ml^−1^ penicillin and 100 *μ*g ml^−1^ streptomycin at 37°C with 5% CO_2_.

Human coronavirus HCoV‐OC43 (ATCC VR‐1558) was cultured in HCT‐8 epithelial cells in RPMI‐1640 with 5% FBS, 100 IU ml^−1^ penicillin and 100 *μ*g ml^−1^ streptomycin for 7 days at 33°C. HCoV‐OC43 was harvested by aspirating the RPMI‐1640 culture media and centrifuging (3000 ***g***, 4 min) to remove cell debris. The virus stocks were stored at −80°C before use. All experiments were performed within a Biosafety Level 2 laboratory.

### Stability of HCoV‐OC43

A layer of either magnesium carbonate chalk (0·1 g; FrictionLabs, Denver, CO), calcium carbonate chalk (0·2 g; Heiltropfen Lab, London, UK) or a chalk blend (1 : 1 magnesium carbonate: calcium carbonate, 0·2 g) was deposited onto 25 cm^2^ PVC PUR plastic swatches.

Aliquots of 200 *μ*l of the model human coronavirus HCoV‐OC43 (4·79 log_10_ TCID_50_ per sample) was transferred via 10 *μ*l droplets to the surface of the chalk and incubated at room temperature for 0, 1, 5, 10, 30 and 60 min. No virus (chalk with 200 *μ*l cell culture media) and no chalk (200 *μ*l HCoV‐OC43) controls were included.

Infectious virus was recovered in 3 ml phosphate buffered saline (PBS; Lonza, Basel, Switzerland) using cotton swabs, vortexed for 30 s and centrifuged at 2500 ***g*** for 10 min to remove excess chalk. The supernatant was syringe filtered (polyether sulfone, 0·45 *μ*m; Fisher Scientific, Loughborough, UK), serially diluted in DMEM supplemented with 5% FBS, 100 IU ml^−1^ penicillin and 100 *μ*g ml^−1^ streptomycin and plated onto BHK‐21 fibroblast cells seeded in a 96‐well format. The plates were incubated at 33°C with 5% CO_2_ for four d before the cells were scored for cytopathic effect. The number of infectious viral particles present on each surface sample (log_10_ TCID_50_ per sample) was then determined according to the Karber method (Ramakrishnan 2016). The detection limit of the assay was 2·33 log_10_ TCID_50_ per sample. The log_10_ reduction of infectious virus for each sample was calculated from the number of infectious virus particles deposited onto the surface (4·79 log_10_ TCID_50_ per sample).

### RT‐qPCR

Chalk powders were inoculated with 200 *μ*l HCoV‐OC43, immediately recovered in three ml PBS as described above and the supernatants harvested. The chalk pellet was resuspended in a further three ml PBS and three cycles of vortexing for 30 s and centrifuging at 2500 ***g*** for 10 min were performed prior to recovering the supernatant. Resulting supernatants were syringe filtered prior to RNA extraction using the Meridian Bioscience (London, UK) ISOLATE II RNA Mini Kit according to the manufacturers’ instructions. A no chalk control was included.

Total RNA samples were DNase‐treated using the Promega (Chilworth, UK) RQ1 RNase‐free DNase kit prior to reverse transcription using random primers and the GoScript™ reverse transcription system (Promega, Chiworth, UK) according to the manufacturers’ instructions. No reverse transcriptase controls were included, where nuclease free water was included in the reaction mix in place of reverse transcriptase.

RT‐qPCR was performed manually using the Meridian Bioscience SensiFAST™ Probe Lo‐ROX Kit with HCoV‐OC43‐specific primers (forward primer, 5‐AGC AAC CAG GCT GAT GTC AAT ACC‐3; reverse primer, 5‐AGC AGA CCT TCC TGA GCC TTC AAT‐3) and probe ([6FAM]TGACATTGTCGATCGGGACCCAAGTA[TAM]) as described by Owczarek *et al*. (2018). No template and no reverse transcriptase controls were included. PCR was performed at 95°C for 2 min, followed by 40 cycles of 95°C for 10 s and 60°C for 20 s using an Applied Biosystems (Waltham, MA) QuantStudio™ 5 real‐time PCR system. The amount of viral RNA was normalized by comparing to a standard curve of a known quantity of infectious HCoV‐OC43 (8·35 log_10_ TCID_50_ per ml).

### Statistical analysis

Viral infectivity experiments were conducted in triplicate on separate occasions (*n* = 3). RT‐qPCR was conducted with technical triplicates of at least two independent experiments (*n* = 2–3). Distribution of the data was ascertained using the Shapiro–Wilk test (Shapiro and Wilk 1965). Significant differences (*P* ≤ 0·05) in recovered infectious virus (log_10_ TCID_50_ per sample) for each condition were determined by the independent samples Kruskal–Wallis test (Kruskal and Wallis 1952) with multiple comparisons using spss ver. 26 (IBM, Armonk, New York, USA). Significant differences (*P* ≤ 0·05) in viral nucleic acid recovered from chalk supernatants and pellets was determined using the related‐samples Wilcoxon signed rank test (Wilcoxon 1945).

## Conflict of Interest

No conflict of interest declared.
